# NGMA-5. An *in vivo* functional genomics screen to identify novel drivers of lung-to-brain metastasis

**DOI:** 10.1093/noajnl/vdab070.020

**Published:** 2021-07-05

**Authors:** Nikoo Aghaei, Fred C Lam, Ekkhard Kasper, Chitra Venugopal, Sheila Singh

**Affiliations:** 1 McMaster University, Hamilton, ON, Canada; 2 Division of Neurosurgery, Hamilton Health Sciences, Hamilton, ON, Canada

## Abstract

**Introduction:**

Brain metastases, the most common tumors of the central nervous system, occur in approximately 20% of primary adult cancers. In particular, 40% of patients with non-small cell lung cancer develop brain metastasis. As systemic therapies for the treatment of non-small cell lung cancer become increasingly effective at controlling primary disease, patients are ironically succumbing to their brain metastases. This highlights a large unmet need to develop novel targeted therapies for the treatment of lung-to-brain metastases (LBM). We hypothesize that an *in vivo* functional genomic screen can identify novel genes that drive LBM.

**Methods:**

To do this, we developed a patient-derived xenograft (PDX) mouse model of LBM using patient lung cancer cell lines. This PDX model of LBM enables the use of fluorescent and bioluminescent *in vivo* imaging to track the progression of lung tumor and brain metastases.

**Results:**

We have performed an *in vivo* genome-wide CRISPR activation screening to identify novel drivers of LBM. We will derive candidate genes through mouse brain and lung tissue sequencing after mice reach endpoint.

**Expected Area of findings:**

This platform will lead to potential therapeutic targets to prevent the formation of LBM and prolong the survival of patients with non-small cell lung cancer.

**Conclusion:**

To the best of our knowledge, this is the first genome-wide *in vivo* CRISPR activation screen searching for drivers of LBM using a PDX animal model. This study can provide a framework to gain a deeper understanding of the regulators of BM formation which will hopefully lead to targeted drug discovery.

